# Supplementary material for: The First Highly Contiguous Genome Assembly of Pikeperch (Sander lucioperca), an Emerging Aquaculture Species in Europe
Source: Genes (Basel). 2019 Sep 13;10(9):708. doi: 10.3390/genes10090708 (PMC6770990; doi:10.3390/genes10090708)
Supplement: Supplementary file 1 [file genes-10-00708-s001.pdf]

Supplementary File 1: Supplementary Tables
The First Highly Contiguous Genome Assembly of Pikeperch (Sander lucioperca), an Emerging Aquaculture Species in Europe

Table S1: Summary statistics of generated whole genome sequencing data

Platform	Libray name	Insert sizes	Total No. of Reads	Total No. of bp	Mean coverage	Read N50 (bp)	Read mean length (bp)
HiSeq X Ten	Piared-end	470 pb	2,761,296,894	412,813,885,653	375X	150	150
	Nextera mate-pair	2-8 kb	491,748,132	74,253,967,932	66X	150	150
		2-10 kb	473,090,686	71,436,693,586	63X	150	150
PacBio Sequel	–	20 kb	5,258,946	66,522,753,592	60X	16,424	12,735

Table S2: Summary of genome characteristics based on k-mer analysis

	K=17	K=19	K=21	K=31
**Mean k-mer depth (bp)**	367	358	350	318
**Heterozygosity rate (%)**	0.068	0.076	0.085	0.12
**Est. genome size (Mb)**	1006.86	1014.28	1024.35	1039
**Single copy size (Mb)**	533.41	552.45	562.85	645.33
**% Single copy portion**	53	54.5	55	62

Table S3: BUSCO analysis results on assemblies of recently publishes Perciforms fish species

						Vertebrates dataset [%]				Actinopterygii dataset [%]			
**Common name**	**Number of Contigs**	**Contig N50** [bp]	**Release year**	**Genome size** [Mb]	**Repeats content**[%]	**complete**	**duplicated**	**partial**	**missing**	**complete**	**duplicated**	**partial**	**missing**
**Yellow drum**	25,182	50,300	2018	566	13.8	97.91	0.71	1.55	0.54	97.4	2.4	1.33	1.27
**Chinese sillago**	802	2,600,000	2018	645	16.73	97.87	2.61	1.43	0.7	96.64	3.8	1.85	1.51
**Northern snakehead**	29,146	81,400	2017	670,4	18.94	98.07	1.14	1.08	0.85	96.73	2.48	1.76	1.51
**dragonfish**	153,398	6,145	2017	805	19.4	77.1	1.45	16.55	6.34	86.6	2.3	5.7	5.7
**Spotted sea bass**	5,765	31,044	2018	668	20.73	85.0	2.91	2.97	12.03	86.21	3.59	2.69	11.1
**Channel bull blenny**	766	6,330,900	2018	700	27.76	91.8	2.23	2.61	5.41	92.84	2.87	2.21	4.95
**European perch**	100,821	18,196	2018	1,050	33.1	87.08	1.11	9.25	3.67	90.14	2.35	5.25	4.65
**Red sea bream**	1,657	2,896,215	2018	909	31.11	97.76	7.87	0.85	1.39	96.97	7.85	1.44	1.59
**Pikeperch**	1,966	3,000,000	2019	1,065	39.8	97.56	1.07	1.55	0.89	96.27	2.45	1.94	1.79

Table S4: Distribution of repetitive element in the *Sander lucioperca* genome

Repeat element	Total length (bp)	% of genome
SINEs	6,210,727	0.7
LINEs	35,255,815	3.92
LTR elements	16,836,875	1.72
DNA elements	172,995,059	16.47
Simple repeats	34,029,937	3.78
Unclassified	130,880,961	12.54
Total	352,303,085	39.13
